# Potential predictors of type-2 diabetes risk: machine learning, synthetic data and wearable health devices

**DOI:** 10.1186/s12859-020-03763-4

**Published:** 2020-12-14

**Authors:** Paola Stolfi, Ilaria Valentini, Maria Concetta Palumbo, Paolo Tieri, Andrea Grignolio, Filippo Castiglione

**Affiliations:** 1 Institute for Applied Mathematics, National Research Council of Italy, Rome, Italy; 2 Institute of Aerospace Medicine “A. Di Loreto”, Rome, Italy; 3 Research Ethics and Integrity Interdepartmental Center, National Research Council of Italy, Rome, Italy; 4 Medical Humanities - International MD Program, Vita-Salute San Raffaele University, Milan, Italy

**Keywords:** Machine learning, Random forest, Emulator, T2D, Computational modeling, Synthetic data

## Abstract

**Background:**

The aim of a recent research project was the investigation of the mechanisms involved in the onset of type 2 diabetes in the absence of familiarity. This has led to the development of a computational model that recapitulates the aetiology of the disease and simulates the immunological and metabolic alterations linked to type-2 diabetes subjected to clinical, physiological, and behavioural features of prototypical human individuals.

**Results:**

We analysed the time course of 46,170 *virtual subjects*, experiencing different lifestyle conditions. We then set up a statistical model able to recapitulate the simulated outcomes.

**Conclusions:**

The resulting machine learning model adequately predicts the synthetic dataset and can, therefore, be used as a computationally-cheaper version of the detailed mathematical model, ready to be implemented on mobile devices to allow self-assessment by informed and aware individuals. The computational model used to generate the dataset of this work is available as a web-service at the following address: http://kraken.iac.rm.cnr.it/T2DM.

## Background

Type 2 diabetes (i.e. non-insulin-dependent, T2D) is a chronic, multifactorial, metabolic disorder typical of late adulthood characterised by less effective hormone insulin efficiency at lowering blood sugar. The World Health Organization reports that type 2 diabetes accounts for 85–90% of all cases of diabetes in the World [1].

There are many different mechanisms that contribute to the onset of T2D [2], therefore research is focusing on the simultaneous observation of several factors such as metabolic, immunological, genetic, and nutritional drivers. A recent study had pointed out a specific state of inflammation, unique for its characteristics and distinct from the classic inflammatory state, which manifests itself in the presence of a high-calorie diet and “susceptible” lifestyles [3]. The term *metaflammation* well describes this kind of inflammation caused by a high caloric and sugar-rich diet which mainly originates in the visceral adipose tissue [4]. This inflammatory-eliciting insult triggers a cellular response consisting of the release of several intracellular signals and a low levels of cytokines such as Tumour Necrosis Factor-*α* (TNF-*α*), and Interleukin-6 (IL-6) [5]. Moreover, experiments have shown a correlation of these triggers with the inhibition of the insulin signal by phosphorylation of a serine in the Insulin Receptor Substrate-1 (IRS-1) [6]. The result is a malfunctioning receptor unable to bind insulin, turning the cells as insulin-resistant. Summarising, the prolonged condition of a pro-inflammatory response alters the metabolic functions of the adipocytes [7] and, in the long term, causes hyperglycemia and eventually full-blown type 2 diabetes [8].

The scenario just depicted calls for a predictive approach aimed at identifying the metabolic and inflammatory “driving factors”, possibly amenable to being implemented on self-monitoring devices. This has been the main aim of the EU-funded project “Multi-scale Immune System Simulator for the Onset of Type 2 Diabetes” (MISSION-T2D) [9] which has led to the development of a validated multi-level patient-specific model able to integrate metabolic, nutritional and lifestyle data for the prediction of the metabolic and inflammatory processes underlying the development of type 2 diabetes in the absence of familiarity.

## Approach

The mentioned computational model (herein referred to as M-T2D) has been implemented to take into account a set of user input data and to subsequently provide an estimation of the risk to develop a T2D clinical picture.

Setting a definition for the risk of T2D has not been a trivial task. After a few attempts, we decided to combine the level of insulin resistance, the level of inflammatory cytokines, and the pro-inflammatory cell counts. These observables are, among others, used in the introduced mathematical description of the complex interdependencies among metabolites and pancreatic control as well as among adipose tissue components and inflammation.

Upon setting anthropometric parameters such as age, sex, body weight, height, and providing nutritional habits, fitness status and physical activity patterns by the user, the M-T2D calculates the risk of progressing toward a T2D-related state in a predefined time horizon.

Due to the high level of sophistication, M-T2D is quite computationally expensive (a 6-month simulated period takes many hours to run on a current high-performance computing server) and is therefore not a viable solution to perform self-monitoring and assessment on mobile devices. Because of this limitation, we constructed an approximation, namely a surrogate model being able to forecast the output of the model M-T2D with a reduced computational effort. The need for reducing the computational burden of a simulation tool occurs in many research fields. For instance, [10] proposed a statistical model for computer output being interested in the assessment of the computer code and the identification of the most significant predictors to efficiently design experiments. The authors in [11] investigated the same issues considering a Bayesian approach based on Gaussian processes. The study in [12] proposed a spatio-temporal neural network as a surrogate model for a particular type of chemical process, namely the polymerization reactor. The Gaussian process has been applied in [13] to approximate spatio-temporal processes while [14] used a Gaussian process with a modified before approximate dynamic processes in hydrology. For an up to date review regarding approximated models and techniques for complex processes, the interested reader can refer to [15].

The aim of this work is two-fold: (1) to provide an approximation of the final state of M-T2D via surrogate model at initial conditions out of the experimental design, and, (2) to analyze simulated data to assess the parameters’ value of the simulator used to carry on simulations. To this end, we apply Random Forest, a powerful Machine Learning (ML) algorithm, with finest fitting performances when dealing with complex data-generating processes.

ML is becoming a popular and efficient approach to evaluate multidimensional longitudinal health data in different fields of medical research. Examples of this kind of studies include the diagnosis of asymptomatic liver disease [16], the prediction of opioid dependence [17], the evaluation of sociodemographic determinants of health status in aging [18], the prediction of the mobility of medical rescue-vehicles [19], forecasting adverse perioperative outcomes [20], the measure of caloric intake at the population level [21], the personalisation of oncological treatment in radiogenomics [22], the determination of features of systolic blood pressure variability [23], the identification of clinical variables in bipolar disorder [24] and, interestingly, a specific interest in uncovering potential predictors of diabetes (type 1 and 2) using large set of data [25–32]. ML can also support global efforts in various fields of epidemic outbreaks of infectious diseases, developing up-to-date text and data-mining techniques to assist COVID-19-related research, especially by developing drugs faster (screening and detecting antibody virus interactions and detect viral antigens), understanding viruses better, mapping where viruses come from, and hopefully predicting the next pandemic [33, 34]. ML may offer accurate results with fewer requirements if compared with traditional mathematical modeling and it is often used to extract harder-to-detect knowledge from unstructured data. ML models are particularly useful in settings where the input is represented by the enormous amount of diagnostic data whereas the output consists in predictive therapeutic options. At variance with the classical application of ML methods, in the present work which deals with the prediction of the risk of T2D, we use Random Forest to “approximate” M-T2D. For this purpose, the training set consisted of a large number of virtual (i.e. simulated) subjects experiencing different lifestyle conditions. The ML-derived surrogate model can recapitulate the simulated outcomes, thus computing the risk index with a significantly smaller computational effort, therefore allowing, as anticipated, to be computed in real-time on mobile devices.

Advances in wearable devices, computational power, and safe communications are permitting the evolution of precision medicine that could facilitate the development of personalized treatment of diabetes risk of each patient on an individual basis [35]. The accomplishments presented here are thus better valued looking at the great development of self-monitoring systems nowadays embedded in portable communication devices which open up to the application of predictive tools in health care [35]. Such predictive tools integrated with wearable devices, could feed their model-predictive alarm set and control systems with monitored signal data to adapt to the in vivo changes of the metabolic state of the user. The computational cheapness of the surrogate model proposed would then allow using data coming from wearable devices, as soon as they are measured [36], providing, therefore, a real-time calculation of health indicators, whose evaluation would otherwise be unfeasible.

## Methods

In this section we first describe the computational model M-T2D and then we detail the experimental design used to generate the data. Such description is necessary to understand the data analysis that is carried on in the next section.

### The computational model

The whole-body multi-scale computational model for fuel homeostasis M-T2D [37] describes the metabolic, hormonal and inflammatory changes due to exercise sessions and food ingestion [38]. It consists of the combination of many ordinary differential equations and an agent-based model unified into a multi-scale simulation tool.

The metabolic physiology-based sub-model of M-T2D consists of an extended formulation of [39] to describe fuel homeostasis in response to a session of physical exercise. It incorporates the hormonal model inspired by [40] in which both glucagon and insulin are represented and glucose regulation is achieved by altering the balance between the two. Concerning the original model in [39] and with the aim of achieving greater generalization and user-customization, M-T2D provides an enhanced description of the physical exercise similar to that in [41] and [42]. In particular, we used a “relative” (rather than fixed) exercise intensity as well as the estimation of functional capacity in relation to age, sex, anthropometric characteristics, and current fitness status [37]. Moreover, M-T2D includes oxygen consumption and the dynamics of epinephrine as directly dependent on the relative exercise intensity to modulate hormones and metabolites responses to different exercise modalities (e.g. cycling, walking, running, stepping). For what concerns the description of the physiological changes due to food ingestion, stomach emptying, and absorption of macronutrients monomers in the gut [38] we follow the work in [43] and [44]. The description of the dynamics of alanine and triglycerides from proteins and fats ingestion, respectively, needed the settings of proper parameters, since the model in [43] is limited to the description of glucose dynamics. Insulin resistance or insulin-deficient states leads to a reduced response of tissues, such as the skeletal muscle, liver, and adipose tissue, to insulin, therefore M-T2D also implements the effects of insulin resistance on the glucose uptake by peripheral organs [45]. Besides that, in modeling fasting plasma glucose concentration we took into consideration factors depending on dietary habits, physical activity, and inflammation. These factors contribute in different ways to increase or diminish the blood sugar level. The glycemia (i.e. presence of glucose in the blood) rises due to unhealthy eating habits, leading to inflammation. Also, it decreases if the patient does physical exercises.

All together, M-T2D includes several models: (1) a model of energy balance and weight gain/loss is added in [45], based on the equations provided by [46] and [47]; (2) the emergence of the inflammation is described as the result of adipose mass increase which, in turn, is a direct consequence of a prolonged excess of high-calorie intake [48]; (3) to better describe lifestyle, we include a previously published model of physical exercise [37]; (4) to counteract the inflammatory scenario, the presence of anti-inflammatory mechanisms promoted during exercise by skeletal muscle has been considered, based on a previous published study [49]; (5) finally, to describe the inflammatory status of the subject, M-T2D merges the metabolic model with a general-purpose simulator of the immune system [50], a modeling framework used to study different human pathologies [51–53], specific aspects of the immune response [54, 55] and also aspects of non-human immunity [56].

### The generation of synthetic data

Simulated trajectories of the dynamic metabolic model M-T2D starting from different initial conditions (i.e. anthropometric features, physical activity patterns and dietary habits) corresponding to different virtual subjects have been generated by varying the parameters in Table 1. The total number *m* = 46,170 is thus the product of the following terms ($|\cdot |$ indicates the cardinality of the set):

1 $$\begin{aligned} m = |S| \cdot |A| \cdot |W| \cdot |H| \cdot \left( 1 + N_{\mathrm{PA}} \cdot |D_{\mathrm{PA}}| \cdot |I_{\mathrm{PA}}| \right) \cdot |C_{\mathrm{ME}}| \cdot |P_{\mathrm{ME}}| \cdot |F_{\mathrm{ME}}|. \end{aligned}$$

**Table 1:** The different virtual subjects have been generated by varying the parameters in this table and corresponding to 46,170 different initial conditions

Anthropometric measures	
Sex $S\in \{female, male\}$	
Age $A\in \{28, 38, 48, 58, 68\}$	
Weight $W\in \{ underweight, normal, overweight \}$	
Height $H\in \{ short, average, tall \}$	
Physical activity	
Number of sessions per week $N_{\mathrm{PA}} \in \{ 0, 1, 2, 3\}$	
Duration (mins) $D_{\mathrm{PA}} \in \{ low=30, medium=60, high=90 \}$	
Intensity (% of $\hbox {VO}_{\mathrm{2max}}$) $I_{\mathrm{PA}} \in \{ low = 40, high = 60 \}$	
Food intake (3 meals per day, breakfast, lunch, dinner)	
Carbohydrates (grams) $C_{\mathrm{ME}} \in \{ low, med, high \}$	
Proteins (grams) $P_{\mathrm{ME}} \in \{ low, med, high \}$	
Fats (grams) $F_{\mathrm{ME}} \in \{ low, med, high \}$	

Low/medium/high quantities of carbohydrates, proteins, and fats are computed taking into account the balance of calories between the meal and the total daily energy expenditure (TDEE) [45]. In details, TDEE is the result of the sum of Resting Energy Expenditure (REE), Activity Energy Expenditure (AEE) [57] and Thermic Effect of Food (TEF) [58]. We implemented the equations by Mifflin and coworkers in [46] to estimate the REE considering weight, height, age, and sex. We determine the AEE based on the intensity, duration, volume of oxygen consumed, and the number of sessions of the exercise as in [45]. Finally, the TEF is the amount of energy expenditure that occurs after eating, due to the cost of digesting and processing food and represents about 10% of the calories due to meal ingestion [58]. The resulting TDEE represents the number of calories that have to be ingested to have a balance among energy intake and expenditure. In our calculation, these calories are somehow arbitrarily yet realistically split between breakfast (25%TDEE), lunch (45%TDEE) and dinner (30%TDEE). Furthermore, for each meal, we divided the caloric content of the meal in calories from carbohydrates, proteins, and fats equal to 50%, 20%, and 30%, respectively. Finally, to convert calories to grams we used the Atwater general factor system [59]. These “standards” or average values of grams for carbohydrates, proteins, and fats are used as reference values (median or ’med’ value). Simple multiplications to the constants 0.8 and 1.5 are used to fix ’low’ and ’high’ quantities of the food intake description given above. The complete patient specification of the initial condition of the simulation is thus given as a string vector. For instance, the initial condition specified by the string female 28 obese tall 2 60/40 low/high/low corresponds to a 28 years old female subject, tall and obese, who exercises twice a week (sixty minutes each time with an intensity of 40%$\hbox {VO}_{2\max}$) and who follows a diet consisting in a low amount of carbohydrates and fats but rich in proteins. So in general we indicate the vector corresponding to the initial condition as follows:

2 $$\begin{aligned} {\varvec{x}}=\left [ S,A,W,H,(N_{PA},D_{\mathrm{PA}},I_{\mathrm{PA}}),(C_{\mathrm{ME}},P_{\mathrm{ME}},F_{\mathrm{ME}}) \right ]. \end{aligned}$$

Simulations’ outputs were analyzed based on the following variables which are deemed the most significant to calculate the risk of developing T2D: Glucose BaseLine (*GBL*, namely the fasting glucose concentration), Body Mass Index (*BMI*), and Tumor Necrosis Factor-$\alpha$ (*TNF*) as measured in the adipose tissue compartment. The execution of M-T2D starting from the initial condition ${\varvec{x}}$ generated a complete trajectory of these variables with a time resolution of ten seconds. However, since we are interested in analysing the condition of the virtual subject only at the end of a specified period of 6 months, these measures are taken after 6 months of routinely and uninterrupted physical activity and diet patterns as specified (among the other things) in ${\varvec{x}}$. Formally,

3 $$\begin{aligned} {\varvec{y}}=\left[ BMI(t),GBL(t),TNF(t)\right] \end{aligned}$$

where *t* is 6 months. The set $\{ ( {\varvec{x}}^{(k)}, {\varvec{y}}^{(k)} ) : k=1,\dots ,m \}$ is used as a training set for the development of a statistical model able to recapitulate, given ${\varvec{x}}$, the dynamics of the computational model and to predict the risk of developing T2D over a time horizon of 6 months. In other words, our goal was to find a statistical/ML model (which should not be confused with the computational model M-T2D) able to predict the *dependent variables*, namely ${\varvec{y}}$ given a set of *regressors/predictors* ${\varvec{x}}$, that is, the initial conditions defining the virtual subject and her/his lifestyle. The new statistical model is, therefore, a *surrogate model* of M-T2D whose role is only to forecast the T2D risk after 6 months for given initial conditions that were not considered for the construction of the synthetic data. The main reason for finding such ML model is that the complexity of M-T2D requires a significant computational effort to run, whereas a statistical model, once trained, provides a real time solution of computing ${\varvec{y}}^{(i)}$ given ${\varvec{x}}^{(i)}$ allowing a fast generalisation to cases other than those in the training set $\left\{ {\varvec{x}}^{(k)}, {\varvec{y}}^{(k)} \right\} _{k=1,\dots ,m}$.

## Results

In order to be a viable solution to the given time and computational restriction, the model should have the following characteristics: good fitting performance in predicting the expected behaviour at time *t* of the output variables given the input variables at time $t_{0}<t$, where $t-t_{0}=6$ months; usability of the results in analysing the impact of each regressor on the output; computational inexpensiveness in order to be implemented on wearable devices. To this end, we adopted a data driven approach over the simulated patterns, in particular, using the notation introduced in the previous section, the ML model has been constructed and validated by using the initial conditions ${\varvec{x}}$ of the regressors as input variables and the dependent variables ${\varvec{y}}$ as output variables.

In this section, we first carry on a preliminary analysis to understand the quantitative characteristics of the data and the need to choose Random Forest as ML algorithm among many others.

### Preliminary analysis

**Fig. 1:**
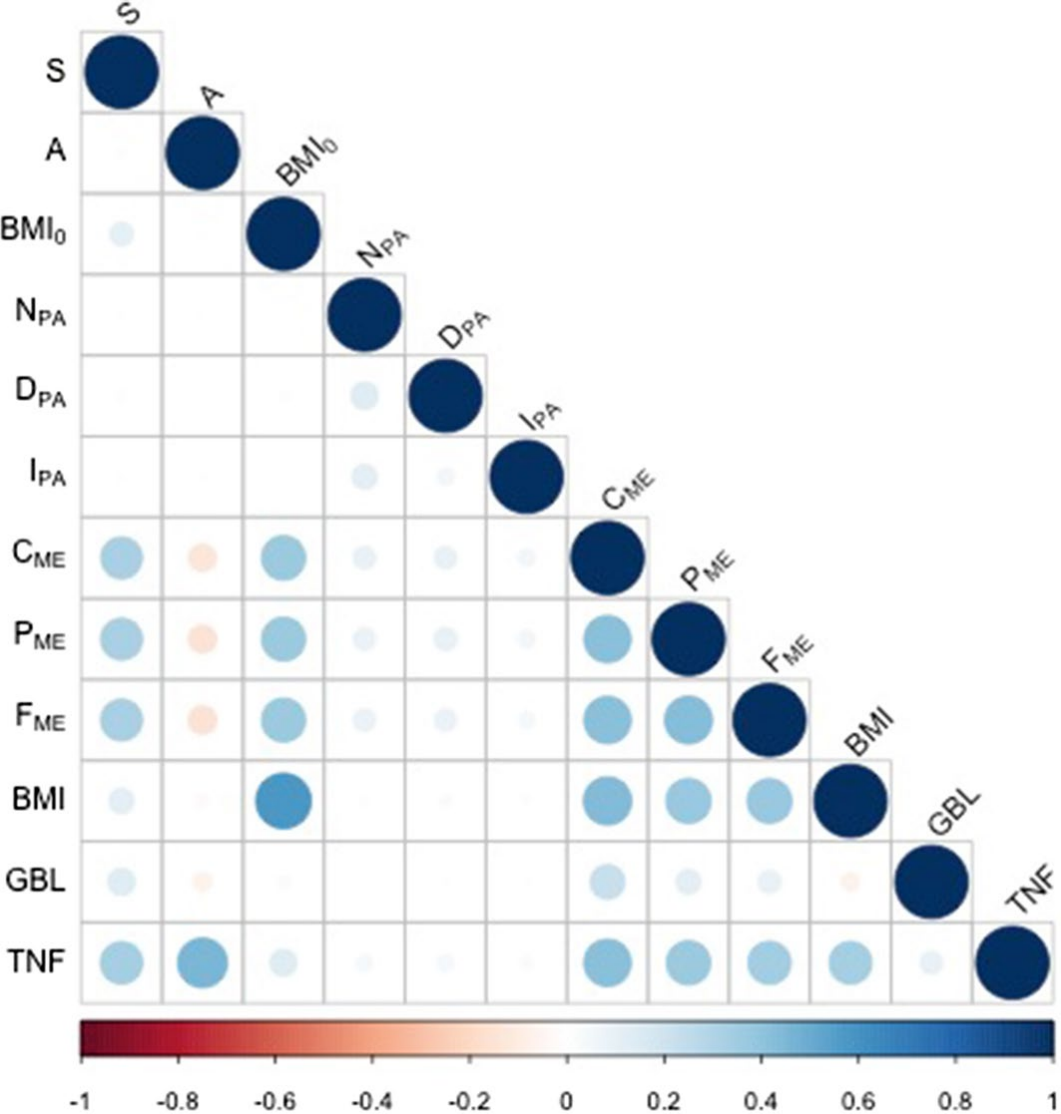
The dots represent the correlations between each couple of variables: the bigger the dots the higher the correlation in absolute value. Numerical value follows the color code in the bar.

Figure 1 shows the correlations among variables. In particular, the dots in the boxes represent the sample Pearson Correlation Coefficients $\rho _{ij}$ between $x_{i}$ and $x_{j}$, namely,

$$\begin{aligned} \rho _{ij}= \left[ \left( m-1\right) s_{i}s_{j} \right] ^{-1} \cdot \sum _{k}^{m}\left[ \left( x_{i}^{(k)}-\mu _{i}\right) \left( x_{j}^{(k)}-\mu _{j}\right) \right] , \end{aligned}$$

where $s_{i},s_{j}$ are the standard deviations and $\mu _{i}, \mu _{j}$ are the mean of variables $x_{i}$ and $x_{j}$ respectively. Their significance is indicated by both the size of the dot (larger means higher significance) and the color (the actual value).

**Fig. 2:**
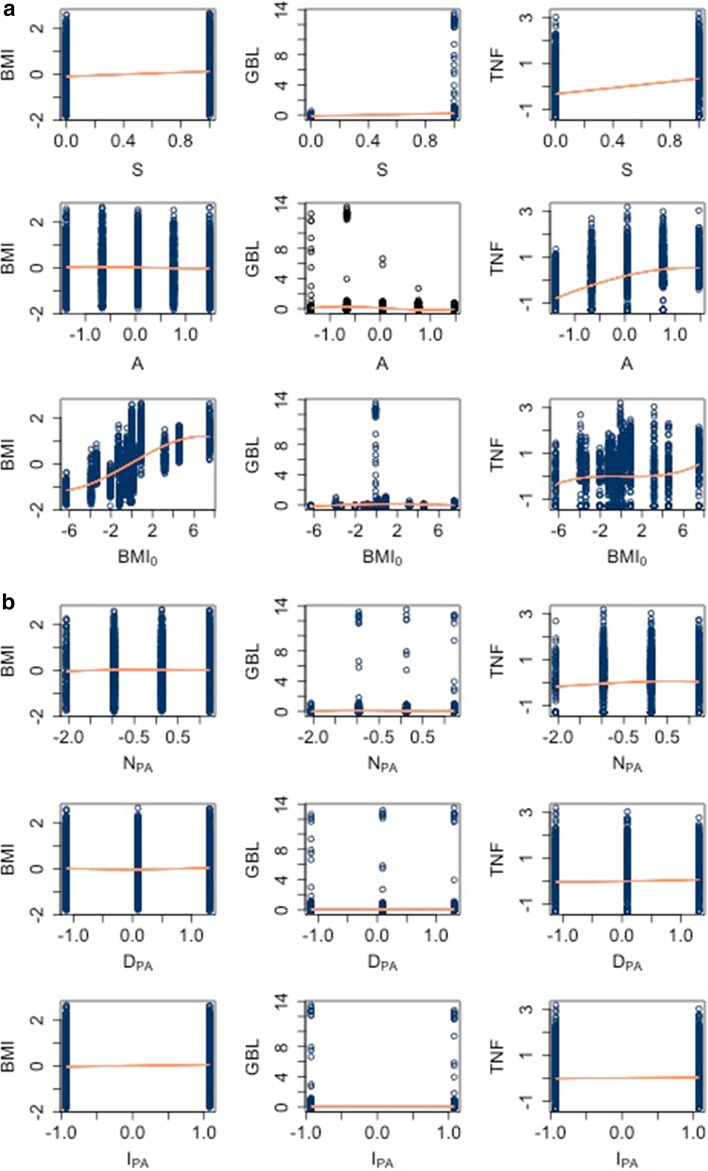

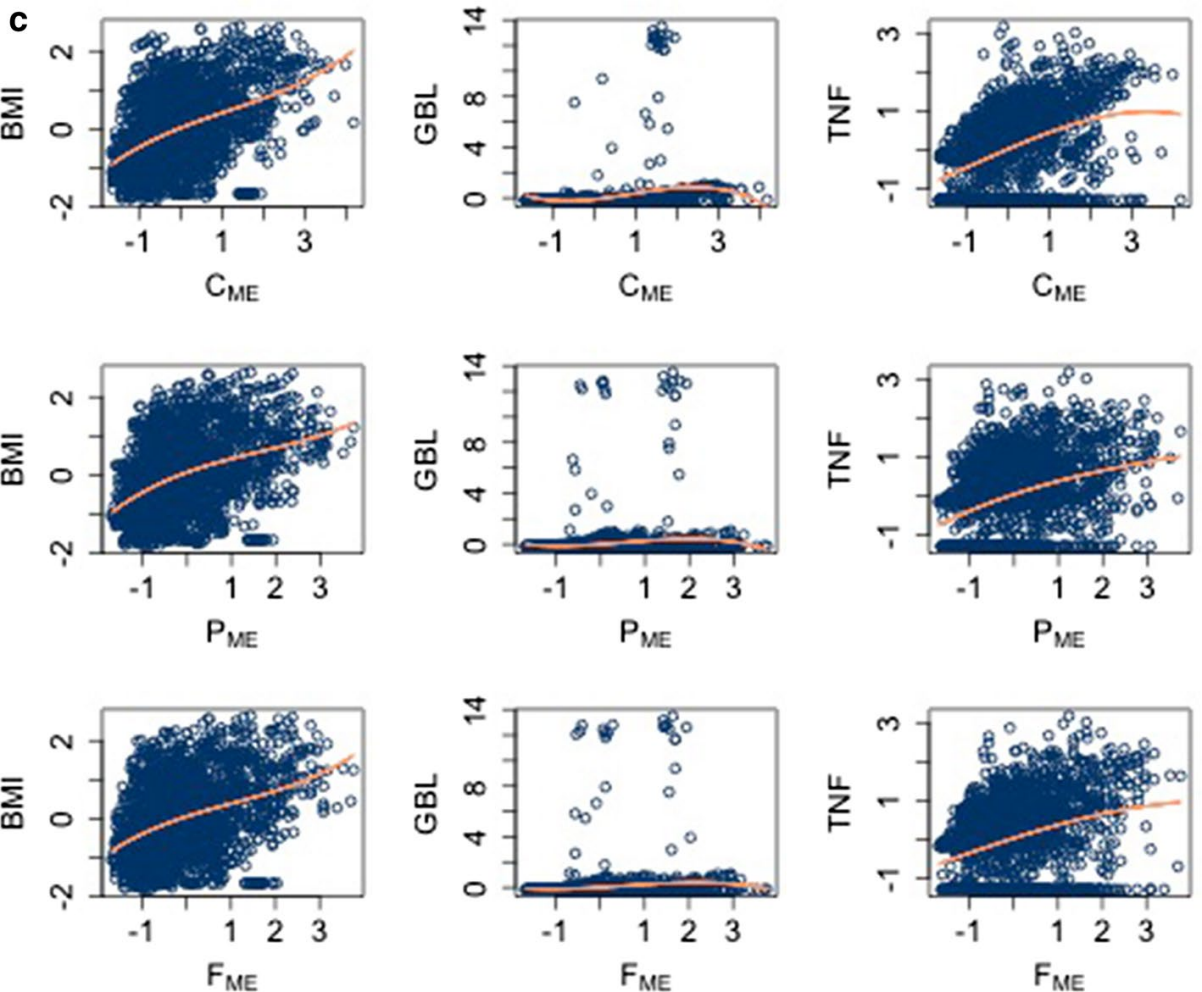
Scatter plots of the independent versus the dependent variables, together with a polynomial fit in orange

In Fig. 2 we report the scatter plots of the regressors and the dependent variables together with the fit (in orange; note: a poor fit, that is, a lack of dependence between the two variables appears as a horizontal or vertical orange line).

Figures 1 and 2 allow identifying critical key features of the dataset. We noticed that there are non-linear dependencies between the output variables and the regressors (e.g. scatter plot of $BMI_{0}=W_{0}/H^2$ in the third row). This observation was expected and, given the high level of complexity of the process generating the data, it suggests that a non-linear ML model should be considered rather than a linear one. Moreover, the variables related to the diet ($C_{\mathrm{ME}}, P_{\mathrm{ME}}, F_{\mathrm{ME}}$, cfr. correlation plot in the middleboxes next to diagonal) do appear strongly correlated. However, these correlations are “spurious” because the corresponding variables depend linearly on another variable indicating the amount of calorie intake (already discussed in the previous section and [45]). Lastly, the correlation plot shows that the output variables *BMI*, *GBL* and *TNF* are correlated, see for instance the dot in position $TNF-BMI$.

All of above observations strongly suggest that a multivariate model is the appropriate choice in the attempt to construct a ML model recapitulating the data. Specifically, we are looking for a statistical model defined as

4 $$\begin{aligned} {\varvec{y}}=\psi \left( {\varvec{x}}\right) +\varvec{\epsilon }, \quad \varvec{\epsilon }\sim {\mathcal {N}}_{3}\left( {\varvec{0}}, \varvec{\Sigma }\right) , \end{aligned}$$

where ${\varvec{x}}$ and ${\varvec{y}}$ are the vectors of regressors and dependent variables respectively, ${\mathcal {N}}_{3}$ is a Gaussian in ${\mathbb {R}}^3$ with zero mean and covariance matrix $\varvec{\Sigma }$, and $\psi \left( \cdot \right)$ is a function to be determined. We tested several statistical models and compared their forecasting performance. We started from the simplest, namely, the linear regression model. Even though preliminary results already prove its unfit, it is interesting to quantify the error made by the linear model. Successively, we tested a few non-linear models, specifically, polynomial regression models of orders 2, 3, and 4. Finally, we tested the random forest algorithm [60]. To investigate the performances of each of the above models, we divided the dataset into a train set consisting of 2*m*/3 data points used to estimate the parameters of the models and the remaining *m*/3 data points in the test set used to assess the predictive performance of the model.

**Fig. 3:**
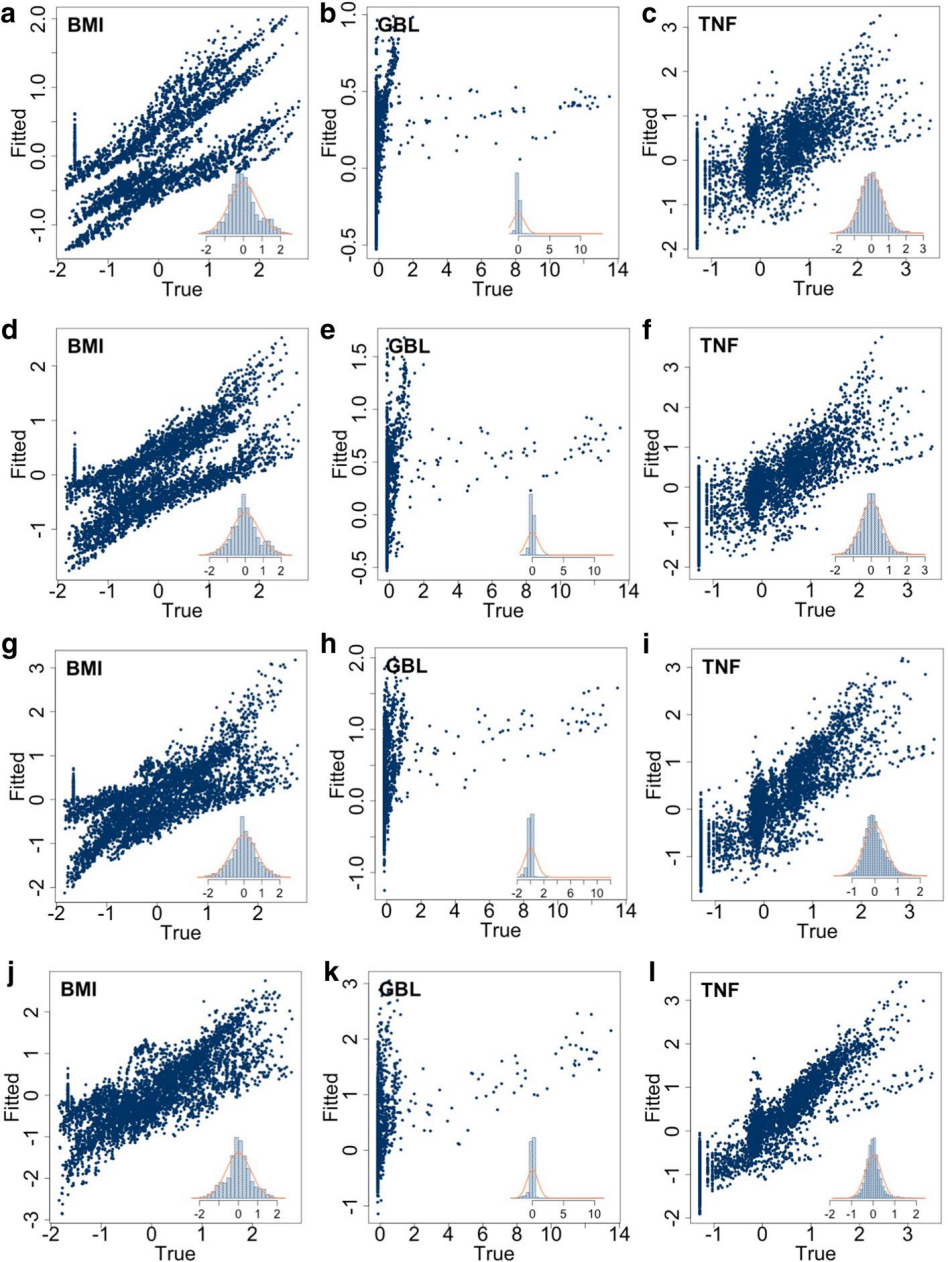

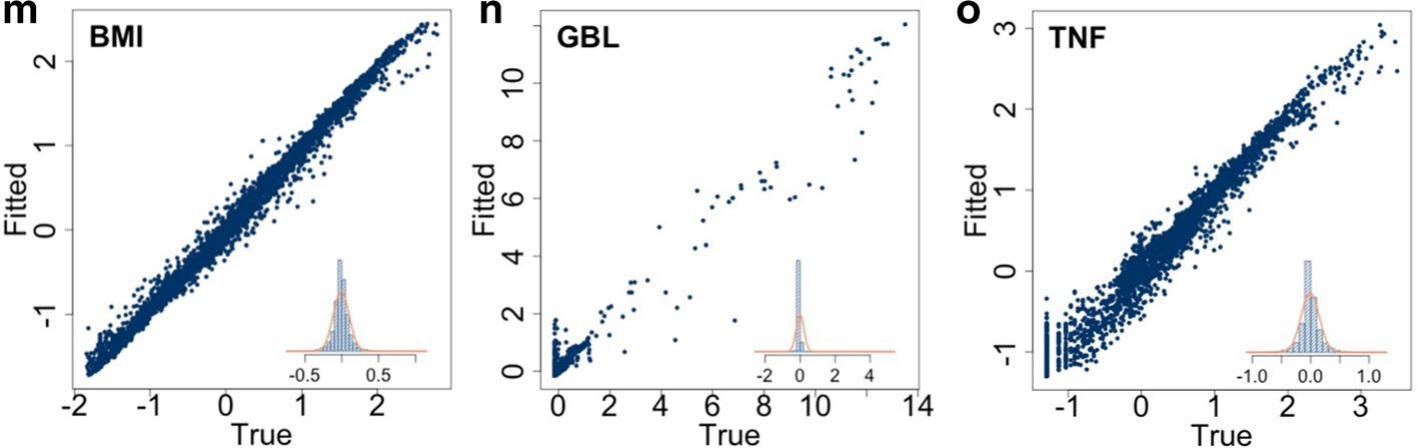
Each row shows the out-of-sample (i.e. in the test set) scatter plots of the true and fitted (i.e. predicted) values of the variables specified in each panel’s caption (from left to right, *BMI*, *GBL* and *TNF*). Inset plots show the histogram of the out-of-sample residues’ (i.e. the prediction error). The last row shows that multivariate random forest performs better predictions when compared to the linear or polynomial regression

Results are shown in Fig. 3. Each row, corresponding to one of the models considered shows the out-of-sample (i.e. computed on the test set) scatter plot of the true versus the predicted values. The linear regression model obtained by defining $\psi$ in Eq. (4) as a linear combination of the regressors, was not able to describe the behavior of none of the dependent variables. Indeed, all scatter plots in Fig. 3a are far from the $y=x$ line. Interestingly, the scatter plots of *BMI* (leftmost panel) and of the *TNF* (rightmost panel) suggests that the linear model does partially capture these variables’ dynamics, indeed despite an unwanted very large variability in the predicted value, a positive correlation between predicted and true values is observed. Conversely, results shown in the middle panel in Fig. 3a pertaining the *GBL* suggest that the linear regression model fails in this case because there is no evident correlation between the true and the fitted values. The result confirms that there is a non-linear structure among ${\varvec{x}}$ and ${\varvec{y}}$ and hints to the use of non-linear models. Figure 3b–d are related to the polynomial regression models of degree $d=2$, 3 and 4 respectively, obtained by defining $\psi$ in Eq. (4) as a polynomial of order *d*. From the plots, it is clear that *BMI* (leftmost panel) and *TNF* (rightmost panel) are only partially described by these models because the scatter plots show large variation in the predicted value hence the use of polynomial models does not improve significantly when increasing *d*. Likewise the linear model, the middle panels in Fig. 3b–d of true versus predicted *GBL* fails to show a clear correlation hence leading to the conclusion that the polynomial structure is also not appropriate.

We then decided to assess other ML approaches, namely decision trees and random forest, based on a tradeoff among forecasting performance, the usability of the results and computational effort required.

### Decision trees and random forest

In statistics/ML, decision trees are powerful tools when dealing with data coming from a complex process with a large number of degrees of freedom, both for regression and classification purposes. The main idea of such tools is to find binary splits, of the form $X_{i}\le c$ and $X_{i}>c$, called *splitting rules*, to divide the dataset into hyper-rectangles being as homogeneous as possible in terms of dependent variables. Homogeneity is measured as the mean square error in the case of regression trees or as Gini index in the case of classification trees. The first node of a decision tree is called *root*, the internal nodes generated by splits are simply called *nodes* and the terminal unsplit are called *leaves*. Each node *k* including the root, is associated to the splitting rule parameters $\theta _{k}=\left( X_{i}, c\right)$ where $X_{i}$ is the splitting predictor and *c* is the splitting value; each leaf *l* is instead associated to dependent variables’ data points $\mu _{l}$ as their mean values in case of regression trees and the most observed value in the case of classification trees [61]. The structure of the tree ${\mathcal {T}}$ is intended to be the whole set of parameters $\theta _{k}$ and $\mu _{l}$, for $k=1,\dots ,K$ and $l=1,\dots ,L$ where *K* and *L* are the total numbers of nodes and leaves respectively.

The major drawback of this regression/classification tool is the high variability characterizing the output, meaning that several trees constructed over the same dataset could produce significantly different outputs. Research has addressed this issue by considering *ensemble methods*. These are methods that generate multiple outputs using the same algorithm but starting from different random initializations.

*Random forest* introduced in [60] is one of the most well-known and powerful regression/classification ensemble method. The general idea of this algorithm is to construct a forest of decision trees and to define the output to be either the mean of all the outputs in the case of regression trees or the result of a majority rule on the output in the case of classification trees.

In detail, the application of the random forest algorithm to predict ${\varvec{y}}$ from ${\varvec{x}}$ with respect to Eq. (4), provides the following formula

$$\begin{aligned} \psi \left( {\varvec{x}}\right) = \frac{1}{N}\sum _{i=1}^{N}{\mathcal {T}}_{i}\left( {\varvec{x}}\right) , \end{aligned}$$

where *N* is the number of decision trees that have been build up and ${\mathcal {T}}_{i}$ is the structure of the *i*-th tree that is the whole set of parameters $\theta _{k,i}$ for $k=1,\dots , K$ and $\mu _{l,i}$ for $l=1,\dots ,L$ as detailed above.

### Learning the parameters of the random forest from synthetic data to predict the risk of T2D

The random forest algorithm has been trained and tested using the scheme adopted for the previous models; the obtained results are shown in Fig. 3e. As clearly shown by the three panels, the multivariate random forest outperforms the previous ones in predicting ${\varvec{y}}$. Indeed the scatter plots of all three variables are aligned on the $y=x$ line indicating a fairly good correlation. Just, a bit of variability is still observed for small values of *GBL* and *TNF*. Looking more in detail, the virtual individuals showing unfit for small values of *GBL* are those having extreme features. An example is given by the virtual individual defined by the following initial conditions ${\varvec{x}}=\left[ \texttt {male, 28, tall, underweight, 1, 60/60, low/low/low} \right]$ that corresponds to a 28 years old male subject, tall (1.91 m) and underweight (65.66 kg), who exercises once a week (sixty minutes each time with an intensity of 60%$\hbox {VO}_{\mathrm{2max}}$) and who follows a diet consisting in a low amount of carbohydrates, fats and proteins, this subject is bordering on anorexia. The lack of knowledge regarding metabolic processes in case of anorexia generates higher variability in simulation’s output that is reflected into a higher unfit of the machine learning algorithm. We focused on the core distribution of the simulation output that is clearly caught up by the random forest algorithm, however, if the interest is toward extreme events quantile regression forest could be a valuable algorithm to analyse the tails of the distribution.

In Table 2 we quantify the error produced by each model as the mean square error (MSE) in measuring the goodness of the fit, in particular, $\mathrm{MSE}_{\mathrm{In}}$ has been computed over the train set (i.e. in sample) while the $\mathrm{MSE}_{\mathrm{Out}}$ has been computed over the test set (i.e. out of sample). As expected, the highest error corresponds to the linear model and it slightly decreases in polynomial regression models when using a higher degree polynomials. Finally, the multivariate random forest regression shows to outperform all other regression methods bringing down the MSE to more than one order of magnitude compared to the polynomial regression. Also to note that the small increase of the $\mathrm{MSE}_{\mathrm{Out}}$ compared to the $\mathrm{MSE}_{\mathrm{In}}$ denotes the absence of overfitting of data.

**Table 2:** In-sample and out-of-sample MSE of all tested models

ML model	$\mathrm{MSE}_{\mathrm{In}}$	$\mathrm{MSE}_{\mathrm{Out}}$
Linear regression	0.6220638	0.6913094
Polynomial degree 2	0.5798217	0.6456507
Polynomial degree 3	0.5016218	0.5675261
Polynomial degree 4	0.4233801	0.493727
Multivariate random forest	0.01991242	0.0276875

## Discussion

**Fig. 4:**
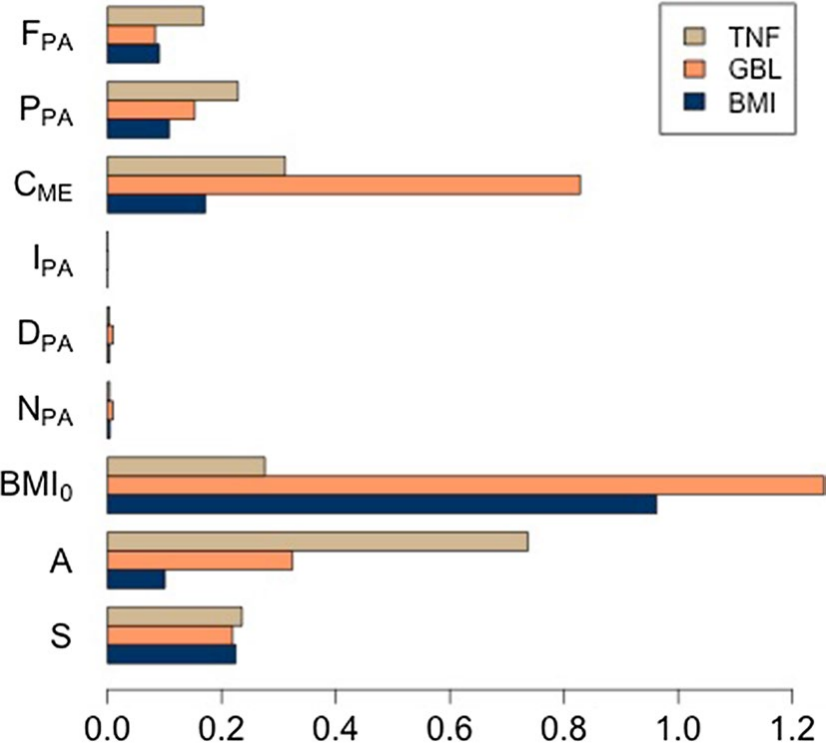
Impact of each input variable ${\varvec{x}}$ on the output ${\varvec{y}}$. Inset plot shows the same data in y-log scale to increase readability (y-scale is in arbitrary units). This plot offers a one-sight readout of the impact of subjects anthropometric measures and lifestyle patterns on the likelihood to progress toward a state of higher risk of development of diabetes

Random forest algorithm showed good fitting performance and it provided a relatively easy interpretation of the data analysis’ results allowing for interesting clinical hints. As first results, we looked at the variables’ importance using a method already described in [62]. In a few words, we measured the impact of each variable on the predictive power of the model, as the difference between the prediction error computed when some noise is added to the predictors and the prediction error computed on the original predictors. Such impact is shown in Fig. 4, where the variables’ importance for each of the elements of ${\varvec{y}}$ are plotted. The impact of some variables appears to be the same for the three variables *BMI*, *GBL*, and *TNF*. Indeed, we observed that the variables related to the physical activity (i.e. $N_{\mathrm{PA}}, D_{\mathrm{PA}}$, and $I_{\mathrm{PA}}$) appear as the less important. This fact points out that better accounting for the physical activity on anti-inflammatory factors as well as on the reduction of glucose baseline already on time horizons smaller than 6 months is required in M-T2D. This is a task that is already ongoing and will be reported in due time [45].

The most important variable for both the *BMI* (grey bar in Fig. 4) and the *GBL* (black bar) is the initial value of the body mass index ($BMI_{0}$). This means that weight plays an important role in determining the glucose baseline thus in the determination of the risk of T2D. As for the remaining variables, we observed that they have a comparable impact on the *BMI*. This is not the same for the glucose baseline or *GBL* index, for which the second most important dependence is with the number of carbohydrates in the diet ($C_{\mathrm{ME}}$). For what concerns the inflammation represented by the level of TNF-$\alpha$ (i.e. *TNF* index, white bar in Fig. 4) the most important dependence is, as expected, the age (*A*) followed in order of importance by $C_{\mathrm{ME}}$ and $BMI_{0}$. This is interesting as it goes along the recently defined concept of *Inflammaging* [63] which joins immune-metabolic processes with age-related diseases in a single, integrated, clinical framework.

**Fig. 5:**
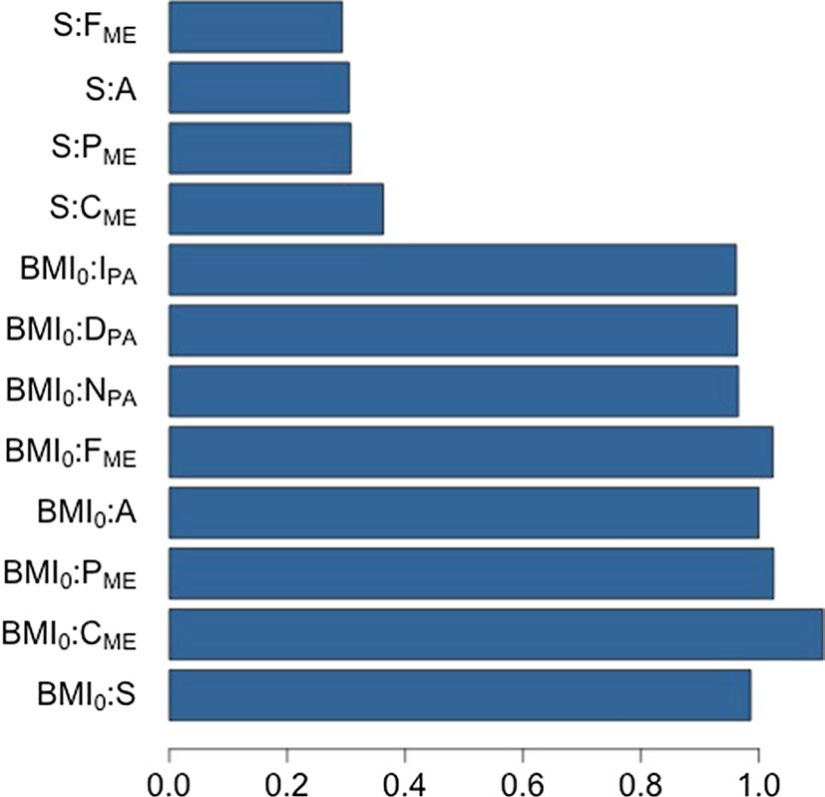
Top twelve pairwise co-influence on ${\varvec{y}}$ calculated by method in [62]

To carry on with the analysis of the relative importance of each input variable, we calculated their influence when taken in pairs. Again, we measure the impact of the couple $\left( x_{i}, x_{j}\right)$ as the difference between prediction error when to $\left( x_{i}, x_{j}\right)$ some noise is added versus the prediction error calculated in the unmodified case [62]. Looking at the pairwise co-influence on ${\varvec{y}}$ in Fig. 5, we noted that the most common of them involve $BMI_0$. This is somehow expected since $BMI_0$ is the most or among the most important variables for all the output variables and both importance analyses are computed using the same methodology.

To overcome any bias coming from this procedure, we considered another method to investigate the variables’ co-influence on ${\varvec{y}}$, namely the *maximal subtree method* [62, 64, 65]. This method is based on the idea that variables that split close to the root play an important role in prediction error, while variables that split next to the leaves do not influence that much the prediction error. To have quantitative method for the idea just explained we need to introduce two concepts [62]: the *maximal* $\nu$-*subtrees* and the *minimal depth*. Given a tree ${\mathcal {T}}$, a $\nu$-*subtrees* ${\mathcal {T}}_{\nu }$ is a subtree of ${\mathcal {T}}$ whose root is split using $\nu$; a *maximal* $\nu$-*subtrees* is a $\nu$-*subtrees* that is not a subtree of a larger $\nu$-*subtrees*. The *minimal depth* $D_{\nu }$ is the distance from the root of ${\mathcal {T}}$ to the root of the closest *maximal* $\nu$-*subtrees*, that is $D_{\nu }$ measures the distance from the root of the first split on $\nu$. The idea explained above can be expressed in terms of the *minimal depth* as follows: the smaller $D_{\nu }$ the higher the impact of $\nu$ on the prediction error.

We apply this method whose result is shown in Table 3, which reports a matrix where the diagonal element $\left( i,i\right)$ represents the normalised (to have a number in the interval $\left( 0,1\right)$) *minimal depth* $D_{i}$ of variable *i*, and the off-diagonal element $\left( i,j\right)$ indicates the normalised *minimal depth* $D_{j}^{i}$ of variable *j* with respect to the *maximal i–subtree* ${\mathcal {T}}_{i}$, that is $D_{j}^{i}$ measures the distance from the root of ${\mathcal {T}}_{i }$ of the first split on *j*. Variables having smaller values on the diagonal are more predictive. Small value on the diagonal element $\left( i,i\right)$ together with small value on the off diagonal element $\left( i,j\right)$ is a sign of significant co-influence on ${\varvec{y}}$ between variables $x_{i}$ and $x_{j}$. This method provides similar results to the one based on the pairwise importance, indeed smaller values of both diagonal and off-diagonal elements correspond to initial $BMI_0$, $C_{\mathrm{ME}}$, *A*, $P_{\mathrm{ME}}$, $F_{\mathrm{ME}}$ and *S* while the variables related to the physical activity shows higher values.

**Table 3:** Pairwise co-influence obtained through maximal subtrees methods. Smaller numbers in the matrix (e.g. $<10^{-1}$) indicate higher influence on ${\varvec{y}}$ of the corresponding pair of variables

	$\hbox {BMI}_{{0}}$	$\hbox {C}_{\mathrm{ME}}$	A	$\hbox {P}_{\mathrm{ME}}$	$\hbox {F}_{\mathrm{ME}}$	S	$\hbox {N}_{\mathrm{PA}}$	$\hbox {S}_{\mathrm{PA}}$	$\hbox {I}_{\mathrm{PA}}$
$\hbox {BMI}_{{0}}$	0.04	0.07	0.07	0.09	0.09	0.13	0.13	0.16	0.22
$\hbox {C}_{\mathrm{ME}}$	0.06	0.06	0.07	0.08	0.08	0.14	0.12	0.15	0.21
A	0.06	0.07	0.07	0.07	0.07	0.13	0.11	0.14	0.20
$\hbox {P}_{\mathrm{ME}}$	0.06	0.07	0.07	0.09	0.08	0.15	0.10	0.13	0.19
$\hbox {F}_{\mathrm{ME}}$	0.06	0.07	0.08	0.07	0.10	0.15	0.10	0.13	0.18
S	0.07	0.07	0.07	0.07	0.08	0.11	0.09	0.12	0.18
$\hbox {N}_{\mathrm{PA}}$	0.08	0.08	0.09	0.08	0.08	0.21	0.18	0.14	0.20
$\hbox {D}_{\mathrm{PA}}$	0.10	0.10	0.13	0.11	0.11	0.31	0.13	0.20	0.20
$\hbox {I}_{\mathrm{PA}}$	0.21	0.15	0.22	0.15	0.15	0.68	0.22	0.24	0.26

As shown in Table 3, we noted that age and diet, taken together, play a significant influence on the outcome ${\varvec{y}}$, that is, on the overall risk of progressing to T2D. The same can be said for gender and diet. Conclusions on the effect of physical activity can not be appreciated at least on a time horizon of 6 months, as we already pointed out when discussing the variables’ importance, while the co-influence of either gender or age with the number of physical activities performed per week has an impact on the risk of T2D larger than the impact of the duration and the intensities of the bouts of exercises.

## Conclusions

Effective prevention of type 2 diabetes onset in the population can be helped by close and regular checks for early detection of signs of progression into the disease. A tool which allows self-assessment based on lifestyle parameters, however approximate, remains a very valuable and beneficial means to increase awareness of the risk of T2D. Nowadays, tools of this kind are within technological reach thanks to the wide-spread use of monitoring devices able to keep track of exercise and dietary patterns and, at the same time, the coming into view of computational methods which estimate the risk of progressing from the healthy (i.e. pre-diabetic) to the disease condition.

The present study shows that it is possible to positively exploit these technologies. Smartphones, tablets, wristwatches and wearable devices are, and increasingly will be, used in everyday life as tools with the potential to foster a proper and healthy life, creating a positive impact on users with an improved effect on the quality of life. Today, the ability to estimate an individual patient’s trajectory risk in real-time remains poor. Knowledge of a patient’s dynamic risk profile may allow physicians to modify targeted and step by step changes in the T2D care plan that will alter the patient’s outcome trajectory [20]. At present, computational tools which exploit the availability of massive data collected by personal assistant devices employing ML techniques are the focus of a great deal of research efforts. Considering recent improvements in healthcare delivery technologies like smartphone applications, device connectivity, artificial intelligence and machine-learning technology there is strong opportunity to reach better efficiency in pre-diabetes and diabetes care, and ameliorate patient involvement in diabetes self-management, which can decrease the surge of diabetes-related healthcare expenditures, paving the way to the future scenario of patient-driven diabetes care in the technology era [66]. Also, this new approach has great potential as a low-cost monitoring tool for nutritional habits and physical activity of different segments of the population, permitting their users to achieve knowledge hardly comprehensible by even the best expert.

In this work, we have shown how a computational model running very complex simulations of realistic multivariate scenarios can be used to feed a machine learning method which demonstrated to perform satisfactorily to predict the risk of T2D using notably less time and computational resources, making it compliant for mobile devices use and for customized and immediate responses to the users. Here we focused on the prediction of the final state of the simulator has given some initial conditions, therefore in the current implementation the ML model provides a 6 months ahead risk of T2D to the users; this time horizon will be extended to predict the whole dynamic of the simulator. This extension, that will be presented in due time [45], will provide the complete dynamic of the variables related to T2D risk, thus becoming a powerful instrument for users as a short- and mid-term assessment tool. In perspective, the ability to link the subject’s parameters with measuring devices such as those in portable communication systems (smartphones and wristwatches) enables the development of health care systems linked in real-time to issue alerts, warnings or simple recommendations to the patient [35]. In the near future, the “real-time” execution of the model, with completely customizable input parameters can be envisaged as a dedicated bioinformatics service, able to provide increasingly personalized healthcare and facilitating self-monitoring.

We conclude by looking at the near future, where we envision at least two avenues of research. A new era of medicine is opening up by combining traditional data from *randomised clinical trials* with new *real-world data*, collected from registries, electronic health records, social media, and wearable devices which produce *real-world evidence*, which can both uncover potential predictors of diabetes or challenge several RCTs data so far collected [32].

A final word should be spent to mention how needed is to open a bioethical debate (beyond, and in respect to, the EU General Data Protection Regulation or any other national regulations) on how to use and secure sensitive health data obtained by wearable devices, at stake, there are ethical questions about practices aimed at monetizing the patients’ data rather than therapeutic quality improvement [67].

## Data Availability

The datasets generated and/or analysed during the current study are available as a web-service at the following address: http://kraken.iac.rm.cnr.it/T2DM.

## Abbreviations

T2D: Type 2 diabetes
TNF-*α*: Tumor Necrosis Factor-*α*
IL-6: Interleukin-6
IRS-1: Insulin Receptor Substrate-1
M-T2D: Multi-scale Immune System Simulator for the Onset of Type 2 Diabetes
ML: Machine Learning
TDEE: Total daily energy expenditure
REE: Resting energy expenditure
TEF: Thermic effect of food
AEE: Activity energy expenditure
S: Sex
A: Age
W: Weight
H: Height
N_*PA*_: Number of physical activity sessions per week
D_*PA*_: Duration of physical activity sessions in minutes
I_*PA*_: Intensity of physical activity sessions
C_*ME*_: Mean carbohydrates intake per day
F_*ME*_: Mean fat intake per day
P_*ME*_: Mean protein intake per day
GBL: Glucose Base Line
BMI: Body Mass Index
BMI_0_: Body Mass Index at time 0
MSE: Mean square error
MSE_*In*_: In-sample mean square error
MSE_*Out*_: Out-of-sample mean square error
